# Risk Factors for Short-Term Lung Cancer Survival

**DOI:** 10.3390/jcm10030519

**Published:** 2021-02-01

**Authors:** Alberto Caballero-Vázquez, José Luis Romero-Béjar, Luis Albendín-García, Nora Suleiman-Martos, José Luis Gómez-Urquiza, Gustavo Raúl Cañadas, Guillermo Arturo Cañadas-De la Fuente

**Affiliations:** 1 Diagnostic Lung Cancer Unit, Broncopleural Techniques and Interventional Pulmonology Departament, Hospital Universitario Virgen de las Nieves, 18014 Granada, Spain; alberto.caballero.sspa@juntadeandalucia.es; 2 Department of Statistics and Operational Research, University of Granada, Avda Fuentenueva, E-18071 Granada, Spain; 3 Faculty of Health Sciences, University of Granada, 18016 Granada, Spain; lualbgar1979@ugr.es (L.A.-G.); jlgurquiza@ugr.es (J.L.G.-U.); gacf@ugr.es (G.A.C.-D.l.F.); 4 Faculty of Health Sciences, Campus Universitario de Ceuta, University of Granada, 51001 Ceuta, Spain; norasm@ugr.es; 5 Department of Didactics of Mathematics, Faculty of Educational Sciences, University of Granada, 18011 Granada, Spain; grcanadas@ugr.es

**Keywords:** epidemiological risk factors, logistic regression, lung cancer, short-term survival, treatments

## Abstract

Background: Lung cancer is typically diagnosed in an advanced phase of its natural history. Explanatory models based on epidemiological and clinical variables provide an approximation of patient survival less than one year using information extracted from the case history only, whereas models involving therapeutic variables must confirm that any treatment applied is worse than surgery in survival terms. Models for classifying less than one year survival for patients diagnosed with lung cancer which are able to identify risk factors and quantify their effect for prognosis are analyzed. Method: Two stepwise binary logistic regression models, based on a retrospective study of 521 cases of patients diagnosed with lung cancer in the Interventional Pneumology Unit at the Hospital “Virgen de las Nieves”, Granada, Spain. Results: The first model included variables age, history of pulmonary neoplasm, tumor location, dyspnea, dysphonia, and chest pain. The independent risk factors age greater than 70 years, a peripheral location, dyspnea and dysphonia were significant. For the second model, treatments were also significant. Conclusions: Age, history of pulmonary neoplasm, tumor location, dyspnea, dysphonia, and chest pain are predictors for survival in patients diagnosed with lung cancer at the time of diagnosis. The treatment applied is significant for classifying less than one year survival time which confirms that any treatment is markedly inferior to surgery in terms of survival. This allows to consider applications of more or less aggressive treatments, anticipation of palliative cares or comfort measures, inclusion in clinical trials, etc.

## 1. Introduction

Lung cancer is the most common cause of neoplasm-related death in both men and women, causing around 1.3 million deaths per year worldwide [1,2,3]. Lung cancer patients experience a wide range of symptoms depending, amongst other factors, on the anatomical structures affected, with the most common being dyspnea, cough, hemoptysis, chest pain, constitutional symptoms, and dysphonia [4,5]. Dyspnea is a very common symptom in advanced lung cancer, being present in 65% of cases at some stage of the disease and having a very marked impact on quality of life [6,7]. Cough, which tends to be caused by intrinsic or extrinsic (adenopathies) obstruction of the trachea and proximal bronchi, is also a common and annoying symptom for lung cancer patients and is often the first symptom to appear [8]. Hemoptysis is present in 7–10% of lung cancer patients and is the cause of death in 3% of cases, being more common in patients with central lesions of the airways than in those with peripheral lesions of the pulmonary parenchyma [9,10]. Chest pain is the most common symptom of lung cancer, being present in approximately 25% of cases at diagnosis and increasing in prevalence as the disease progresses [11]. Constitutional symptoms, including the presence of asthenia, anorexia, and weight loss, generally present in advanced stages of lung cancer and are an important predictor of mortality [12,13]. Dysphonia appears in lung cancer as a result of involvement of the recurrent laryngeal nerve due to metastatic invasion of the mediastinum, mainly the one located in the upper right paratracheal region or in the aortopulmonary window, and generally implies advanced or non-resectable disease [14]. The main treatment options for lung cancer are surgery, radiotherapy, and chemotherapy, either alone or in combination, depending on the stage and histological subtype of the cancer and the clinical situation and general condition of the patient. In advanced stages, or in patients with marked clinical deterioration not susceptible to surgical treatment or chemo- or radiotherapy, palliative care is considered in order to treat the symptoms caused by the cancer and its progression [15,16,17]. The reason for classifying cancer into different groups or stages arises due to the fact that survival is higher in patients in whom the disease is localized than in those in whom the disease can be considered to be advanced. This division of lung cancer into stages helps with treatment planning, gives an idea of the prognosis and allow treatment outcomes to be evaluated [18]. Thus, in general terms, early stage non-small cell lung cancer (stage I and II) is considered to be a good candidate for surgical treatment, with a higher survival rate than for patients in advanced stages (III and IV). The latter benefit from chemotherapy and/or radiotherapy rather than being candidates for surgical treatment, except in the case of stage IIIA, where surgery with prior neoadjuvant therapy can be considered [19,20,21,22]. Non-small cell lung cancer (NSCLC) corresponds to 85% of lung cancers and, unfortunately, up to 80% of cases are diagnosed in advanced stages requiring systemic therapy. The treatment of these patients has made significant progress in recent decades with the emergence of specific mutations targeting therapies (known as targeted therapies) and more recently with immunotherapy, being an increasingly common therapy.

Lung cancer is typically diagnosed in an advanced phase of its natural history, thus resulting in a five-year mortality from diagnosis of 85–90%, with the one-year survival for small-cell carcinoma being markedly lower [23]. In addition, around 80% of patients are inoperable or non-resectable at diagnosis. Consequently, when diagnosed, most cases are not susceptible to curative surgical treatment, with the remaining therapeutic alternatives being mainly chemotherapy, radiotherapy, palliative care, or a combination thereof [24]. Explanatory models based on epidemiological and clinical variables could provide a first approximation of patient survival using information extracted from the case history only, or obtained from non-invasive complementary tests, whereas models involving therapeutic variables must confirm that any treatment applied is worse than surgery in survival terms.

This study has the purpose to identify risk factors and quantify their effect for prognosis for classifying survivals of less than or more than one year for patients diagnosed with non-small cell lung cancer, based on, by means of non-invasive procedures, clinical/epidemiological characteristics at diagnosis and with respect to the treatments received.

## 2. Materials and Methods

### 2.1. Participants

The sample comprised 521 patients diagnosed with non-small cell lung cancer at the Diagnostic Techniques Unit of the Pneumology Service at the Hospital Virgen de las Nieves between 1 January 2011 and 31 December 2016. The mean age of the subjects was 65.2 years (SD = 10.44), 22.3% of them were female, and 38.2% presented survival times of longer than one year.

### 2.2. The Procedure

A retrospective study comprising all patients was carried out. These patients came from the health region for the Universitary Hospital Virgen de las Nieves, which comprises the northern part of Granada and its province, as well as the provinces of Jaén and Almería. Data were obtained from the computerized medical records of each patient and from the provincial tumor registry for Granada. Epidemiological variables, such as age, sex, smoking history and prior neoplasms, and clinical variables, such as the presence of dyspnea, cough, chest pain, hemoptysis, constitutional symptoms, and dysphonia, diagnostic variables related to the way in which the diagnosis of lung cancer was reached in each patient, such as the application of various bronchoscopy techniques (endobronchial biopsy, cytological brushing, or blind puncture), echo-bronchoscopy-guided puncture, ultrasound or CT and PET-CT as an extension and staging study, amongst others, and therapeutic variables, which included the initial treatment administered or applied to the patient upon diagnosis, mainly surgery, chemotherapy, radiotherapy, palliative care, or a combination thereof, were collected. Finally, survival data were collected by obtaining the exact date of death from the electronic records of the regional healthcare system in Andalusia.

### 2.3. Statistical Methods

Logistic regression is one of the statistical tools with the best capacity for data analysis in clinical and epidemiological research; hence, its wide use [25]. According to [26,27] it is a statistical tool for multivariate analysis, which provides information of two types. Firstly, logistic regression provides information of which explanatory variables are risk factors because they involve level changes in the dependent variable. Secondly, this model also provides predictive information because it calculates probabilities of an individual classify in a level response of the dependent variable in relation to the values of the explanatory variables. This allows to build diagnostic tools that once validated can be useful to classify individuals in relation to their response to a pathology according to the risk factors included in the model.

The logit model allows another type of analysis, by means of Odds-Ratio. They are the exponentials of the parameters estimated for each of the risk factors within the model. They provides information about how much is multiplied the advantage of answering a value of the dependent variable versus to another when comparing two categories of the explanatory variable (if it is categorical) or increasing one unit (if it is quantitative).

A binary-response logistic regression model (logit) was used [25,26,27]. Two models were fitted for the response variable that informs regarding the survival time less than or greater than one year: a first model considering clinical/epidemiological data as explanatory variables and a secondary model considering the therapeutic variables as regressors. These models were used to determine which variables caused these patients to have a survival of less than one year. A model containing the effects of the factors, with no interaction between them, was considered to best fit the data. This model was fitted in a stepwise way starting from a constant model, using forward selection to determine whether a variable enters, and backward selection to determine whether it exits, in each step. The goodness-of-fit was compared using the likelihood ratio test, the Hosmer–Lemeshow test and Pearson’s chi-squared test. The statistical significance of the parameters for the variables that enter into each model was evaluated using Wald’s test and the prognosis ratios for each level with respect to the adjacent level were obtained, depending on the possible changes in the explanatory variables considered. Statistical analyses were performed using the program SPSS 19.0.

### 2.4. Ethical Considerations

The study was carried out in accordance with the 1975 Declaration of Helsinki [28] and was approved by the Clinical Research Ethics Committee at the Andalusian Health Service (LUNG CA SURV 2180-N-20). The data were processed in accordance with the provisions of Act 15/1999, of 13 December, on the Protection of Personal Data.

## 3. Results

### 3.1. Description of the Sample

The survival times after a diagnosis of cancer were classified into less than or more than one year. The descriptive analysis of the epidemiological, clinical and therapeutic variables considered, and the survival times, are shown in Table 1.

### 3.2. Explanatory Model with Clinical/Epidemiological Variables

The estimated model for the survival time (SUR) includes the explanatory epidemiological variables age (A) and history of pulmonary neoplasm (PULNEO), as well as the clinical variables location (LOC), dyspnea (DYSN), dysphonia (DYSP), and chest pain (CP), and has the following form:

(1) $${\hat{L}}_{i,j,k,l,m,n}={\hat{B}}_{0}-{\hat{B}}_{E}{\left(E\right)}_{i}-{\hat{B}}_{PULNEO}{\left(PULNEO\right)}_{j}-{\hat{B}}_{LOC}{\left(LOC\right)}_{k}-{\hat{B}}_{DYSN}{\left(DYSN\right)}_{l}-{\hat{B}}_{DYSP}{\left(DYSP\right)}_{m}-{\hat{B}}_{CP}{\left(CP\right)}_{n}$$

i = 1,2,3,4,5; k = 1,2,3; j,l,m,n = 1,2(2)

(3) $${\left(A\right)}_{1}={\left(PULNEO\right)}_{1}={\left(LOC\right)}_{1}={\left(DYSN\right)}_{1}={\left(DYSP\right)}_{1}={\left(CP\right)}_{1}=0$$

The parameters estimated for each explanatory variable in the binary logistic regression model for survival can be found in Table 2 below.

In light of the results of the Wald test (see Table 2), the variables aged more than 70 years (*p* = 0.024), a peripheral location (*p* = 0.010), and the patient presenting dyspnea (*p* = 0.021) and dysphonia (*p* = 0.029) are significant at a population-based level. Consequently, the prognosis change ratio for the levels considered (survival greater than vs. less than one year) was analysed for the variables found to be significant in the model. For the age variable, it should be noted that the prognosis ratio for a survival of less than one year was sevenfold higher for patients aged more than 70 years than for those aged less than 40 years (odds ratio (OR) = 0.140; 95% credible interval [CI]: 0.025–0.770). As regards tumor location, the advantage of surviving for more than one year was almost threefold higher in patients with a peripheral tumor location than in those with a tumor in the central region (OR = 2.509; 95% CI: 1.248–5.044). In the case of patients with dyspnea and dysphonia, the possibility of surviving for less than one year was almost twofold (OR = 0.630; 95% CI: 0.425–0.933) and almost threefold higher (OR = 0.395; 95% CI: 0.172–0.909), respectively, than in patients not presenting these clinical symptoms.

### 3.3. Explanatory Model with Therapeutic Variables

The estimated model for the survival time (SUR) includes the therapeutic treatment (TT) as explanatory variable and has the following form:

(4) $${\hat{L}}_{i,j,k,l,m,n}={\hat{B}}_{0}-{\hat{B}}_{\mathrm{TT}}{\left(\mathrm{TT}\right)}_{i}$$

(5) i=1,2,3,4,5,6,7

(6) $${\left(\mathrm{TT}\right)}_{1}=0$$

The parameters estimated for each explanatory variable in the binary logistic regression model for survival can be found in Table 3 below.

Once again, in light of the results of the Wald test (see Table 3), all treatments considered were found to be significant at a population-based level. Consequently, the prognosis change ratio for the levels considered (survival greater than vs. less than one year) was analysed by comparing each of the treatments with surgery, which is the reference treatment used in the model. In the case of conventional chemotherapy, the prognosis ratio for survival of less than one year was sixfold higher (OR = 0.172; 95% CI: 0.0064–0.465) with respect to surgery, whereas the combination chemotherapy/radiotherapy was almost fourfold higher (OR = 0.267; 95% CI: 0.102–0.699). This ratio was 60-fold higher for palliative care (OR = 0.017; 95% CI: 0.004–0.066), 90-fold higher in the case of chemotherapy combined with palliative care (OR = 0.011; 95% CI: 0.001–0.103), and 11-fold higher in the case of radiotherapy combined with palliative care (OR = 0.084; 95% CI: 0.022–0.324). Finally, in the case of treatments other than those discussed above, this ratio for a prognosis of survival for less than one year was fourfold higher (OR = 0.247; 95% CI: 0.093–0.654) than for surgery.

## 4. Discussion

The aim of this study was to establish models for classifying survivals of less than or more than one year for patients diagnosed with lung cancer, based on clinical/epidemiological characteristics at diagnosis and with respect to the treatments received, as well as to identify which of these variables were actually risk factors and to quantify their effect for prognosis. With regard to the first objective and the clinical/epidemiological characteristics, a model that provides a first approximation of patient survival based on data extracted only from the case history or provided by non-invasive complementary tests was obtained. This model included the epidemiological variables age and history of pulmonary neoplasm and the clinical variables location, dyspnea, dysphonia, and chest pain as predictors for a survival time of less than one year for patients diagnosed with lung cancer at the time of diagnosis. Similarly, with regard to the treatment applied or administered, a model that includes this treatment as a significant element for classifying a survival time of less than one year has been obtained. With regard to the second objective, from a clinical/epidemiological viewpoint, an age of more than 70 years, a central location and presenting dyspnea and dysphonia were all found to be risk factors for a survival time of less than one year. Finally, from a therapeutic viewpoint, it was found that treatment is a risk factor for prognosis of a survival time of less than one year and that any treatment applied is worse than surgery in survival terms.

This study provides models that classify patients of lung cancer in a level of survival less or greater than one year based on modifiable risk factors associated to clinical variables as well as related to the treatment received. However, there are several inherent limitations. First, from a clinical/epidemiological viewpoint, the model estimated for a survival-based classification does not take into account other key variables for this purpose. For example, the diagnosis and cytohistological staging of the neoplasm [28,29], or other chronic comorbidities or conditions that may themselves decrease the survival time, such as arrhythmias, ischemic heart disease, diabetes mellitus, and advanced chronic obstructive pulmonary disease, amongst others [30]. For the therapeutic variables, changes to the oncological treatment in patients with poor tolerance or evolution have not been taken into consideration due to the difficulty in accessing this information. Second, although the models are robust, possible interactions between the clinical/epidemiological risk factors have also not been taken into consideration. Consequently, a study of these interactions and the aforementioned variables would be of interest to develop and validate better models for classifying survival times of less than one year [15,31,32,33]. Finally, the number of patients susceptible to surgery, in other words diagnosed at an early stage, also needs to be increased in order to be able to establish therapeutic groups. This group should be as homogeneous as possible in terms of the number of individuals, although given the unique characteristics of lung cancer in terms of the tendency to diagnose this disease in an advanced stage referred to above. This objective will depend on an improvement in the early diagnosis of lung cancer [34,35].

## 5. Conclusions

In the moment of diagnosis of lung cancer it becomes necessary a good prognosis of the survival time based on the history of the patient. This fact jointly to the knowledge of which is the best therapeutic treatment at this moment could help to mitigate the risk of survival less than one year. Our findings of explanatory models based on epidemiological and clinical variables provide a first approximation of patient survival using information extracted from the case history only. This information allows to consider the application of more or less aggressive treatments, anticipation of palliative cares or comfort measures, inclusion in clinical trials or, in addition, to the important psychosocial and emotional implications that are derived. On the other hand our model based on the different therapeutic treatments confirms that that any treatment applied is worse than surgery in survival terms, and it provides ratios between every couple of treatments for survival. Advancing the fight against short-term survival for all lung cancer patients requires improvements of the models and consequently an ongoing continuous research looking for models with more risk factors involved and/or the interaction between all of them.

## Figures and Tables

**Table 1:** Description of variables.

**Response Variable**	**Level**	**% (*n*)**
SUR = Survival (*n = 521*)	(0) Less than one year	61.8 (322)
	(1) More than one year	38.2 (199)
**Epidemiological Variables**	**Level**	**% (*n*)**
S = Sex (*n = 521*)	(0) Male	77.7 (405)
	(1) Female	22.3 (116)
A = Age (*n* = 521)	(0) <40 years	1.3 (7)
	(1) 41–50 years	6.7 (35)
	(2) 51–60 years	24.0 (125)
	(3) 61–70 years	34.9 (182)
	(4) >70 years	33.0 (172)
SMO = Current or former smoker? (*n* = 521)	(0) Never	12.5 (65)
	(1) Smoker or ex-smoker	87.5 (456)
PACK = Packs per year (*n* = 521)	(0) <15	17.7 (92)
	(1) 15–30	15.4 (80)
	(2) 30–50	31.1 (162)
	(3) >50	35.9 (187)
NOPULNEO = History of NON-pulmonary neoplasm (*n* = 521)	(0) NO	75.8 (395)
	(1) YES	24.2 (126)
PULNEO = History of pulmonary neoplasm (*n* = 521)	(0) NO	97.3 (507)
	(1) YES	2.7 (14)
**Clinical Variables**	**Level**	**% (*n*)**
CP = Chest pain (*n* = 521)	(0) NO	64.9 (338)
	(1) YES	35.1 (183)
DYSN = Dyspnea (*n* = 521)	(0) NO	63.1 (329)
	(1) YES	36.9 (192)
DYSP = Dysphonia (*n* = 521)	(0) NO	93.1 (485)
	(1) YES	6.9 (36)
HAEM = Haemoptysis (*n* = 521)	(0) NO	79.3 (413)
	(1) YES	20.7 (108)
COU = Cough (*n* = 521)	(0) NO	54.5 (284)
	(1) YES	45.5 (237)
CS = Constitutional symptoms (*n* = 521)	(0) NO	66.2 (345)
	(1) YES	33.8 (176)
LOC = Location (*n* = 521)	(0) Central	28.6 (149)
	(1) Peripheral	8.8 (46)
	(2) Central and peripheral	62.6 (326)
**Therapeutic Variables**	**Level**	**% (*n*)**
TT = Treatment (*n* = 521)	(0) Surgery	5.2 (27)
	(1) Conventional chemotherapy	19.4 (101)
	(2) Chemotherapy + Radiotherapy	28.6 (149)
	(3) Palliative care (PC)	13.6 (71)
	(4) Chemotherapy + PC	5.0 (26)
	(5) Radiotherapy + PC	4.2 (22)
	(6) Other treatments	24.0 (125)

**Table 2:** Logit model for survival (clinical and epidemiological variables).

Predictor	B	DT	Wald	*p*-Value	Odd	CI for 95% Odds	
						Lower	Upper
Constant	1.291	0.879	2.157	0.142	3.637		
${\left(A\right)}_{2}$	−0.793	0.919	0.744	0.388	0.453	0.075	2.741
${\left(A\right)}_{3}$	−1.401	0.873	2.575	0.109	0.246	0.045	1.364
${\left(A\right)}_{4}$	−1.632	0.867	3.538	0.060	0.196	0.036	1.071
${\left(A\right)}_{5}$	−1.967	0.870	5.112	0.024	0.140	0.025	0.770
${\left(\mathrm{PULNEO}\right)}_{2}$	1.065	0.587	3.293	0.070	2.901	0.918	9.166
${\left(\mathrm{LOC}\right)}_{2}$	0.920	0.356	6.670	0.010	2.509	1.248	5.044
${\left(\mathrm{LOC}\right)}_{3}$	0.082	0.215	0.144	0.704	1.085	0.712	1.655
${\left(\mathrm{DYSN}\right)}_{2}$	−0.463	0.200	5.328	0.021	0.630	0.425	0.933
${\left(\mathrm{DYSP}\right)}_{2}$	−0.928	0.425	7.766	0.029	0.395	0.172	0.909
${\left(\mathrm{CP}\right)}_{2}$	−0.342	0.202	2.872	0.090	0.710	0.478	1.055

Note: The reference category for the response variable is less than one year. For the explanatory variable age is: <40 years, for the history of pulmonary neoplasm it is: NO, for location it is central, for dyspnea it is: NO, for dysphonia it is: NO and for chest pain it is: NO. A = Age; B = coefficient; DT = Standard deviation; DYSP = Dysphonia; DYSN = Dysnea; LOC = Location; PULNEO = History of pulmonary neoplasm.

**Table 3:** Logit model for survival (therapeutic variable).

Predictor	B	DT	Wald	*p*	Odd	CI for 95% Odds	
						Lower	Upper
Constant	1.253	0.463	7.324	0.007	3.500		
${\left(\mathrm{TT}\right)}_{2}$	−1.758	0.506	12.054	0.001	0.172	0.064	0.465
${\left(\mathrm{TT}\right)}_{3}$	−1.320	0.491	7.224	0.007	0.267	0.102	0.699
${\left(\mathrm{TT}\right)}_{4}$	−4.071	0.692	34.587	0.000	0.017	0.004	0.066
${\left(\mathrm{TT}\right)}_{5}$	−4.472	1.120	15.942	0.000	0.011	0.001	0.103
${\left(\mathrm{TT}\right)}_{6}$	−2.477	0.688	12.964	0.000	0.084	0.022	0.324
${\left(\mathrm{TT}\right)}_{7}$	−1.397	0.496	7.919	0.005	0.247	0.093	0.654

Note: The reference category for the response variable is: less than one year. For the explanatory variable treatment, the reference category is surgery. B = coefficient; DT = Standard deviation; TT = Treament.

## Data Availability

The data presented in this study are available on request from the corresponding author. The data are not publicly available due to ethical.
